# The Theory of Individual Values in the Chinese Population: Psychometric Examination Based on the Revised Portrait Value Questionnaire

**DOI:** 10.1002/pchj.70023

**Published:** 2025-06-16

**Authors:** Xu Wang, Ni Zhu, Mingchen Wei, Shuai Chen, Cheng Guo, Yanling Liu

**Affiliations:** ^1^ Faculty of Psychsology Southwest University Chongqing China; ^2^ Research Center of Mental Health Education Southwest University Chongqing China; ^3^ Chongqing Yubei Qicheng Bashu Primary School Chongqing China; ^4^ Institute of Curriculum and Instruction, Faculty of Education East China Normal University Shanghai China; ^5^ School of Psychology, Center for Studies of Psychological Application South China Normal University Guangzhou Guangdong China

**Keywords:** individual values, measurement invariance, multidimensional scaling, psychometric examination, the Chinese version of Revised Portrait Value Questionnaire

## Abstract

This study aims to assess the validity of the Revised Portrait Value Questionnaire (PVQ‐RR) within the Chinese population to examine the applicability of the 19‐factor Theory of Individual Values in the Chinese cultural context. A sample of 9590 Chinese participants (age range: 11–60; 4360 males) completed the Chinese version of the PVQ‐RR. Psychometric analyses indicated that the Chinese version of the PVQ‐RR consistently measures 19 basic values and 4 higher‐order values. Multidimensional scaling results showed that the circular structure of individual values among Chinese generally aligns with the Theory of Individual Values, but the positions of some values among the 19 basic individual values exhibit strong cultural characteristics. The findings also revealed that the most valued among Chinese people is security‐societal, while the least valued are power‐resources and power‐dominance. This study reaffirms the cross‐cultural consistency of the Theory of Individual Values and provides a reliable tool for assessing individual values among Chinese people.

## 1 Introduction

Schwartz's Theory of Individual Values holds significant influence in the field of values research. From the initial 10 basic individual values model proposed in 1992 to its expansion to the 19 basic individual values model in 2012, this model has been refined and applied over two decades and has been validated in over 80 countries worldwide (Schwartz et al. 2012), thereby proving its cross‐cultural applicability. Although several studies have been conducted with Chinese samples within this theoretical framework (Heim et al. 2018; Liu et al. 2021; Maercker et al. 2015; Xie et al. 2023), systematic validation results of the Theory of Individual Values in Chinese populations remain inconsistent (Heim et al. 2018; Jin et al. 2019), with a paucity of comprehensive psychometric examinations of the most recent Revised Portrait Value Questionnaire (PVQ‐RR). The PVQ‐RR can be used to measure individual values of persons aged 11 and above who have not received Western education (Schwartz 2012). The current study therefore seeks to examine the applicability of the PVQ‐RR in the Chinese cultural context through extensive sampling across different gender and age groups to explore both theoretical and practical implications of the Theory of Individual Values in Chinese culture.

### 1.1 The Theory of Individual Values

This theory has been meticulously reviewed by many researchers (Sagiv et al. 2017; Schwartz 1992, 2006; Schwartz et al. 2012; Schwartz and Sortheix 2018), and this study provides a brief overview.

Individual values are defined as broad goals that serve as guiding principles in individual lives (Schwartz 1992). Schwartz (1992) proposed that there are three universal motivations for the survival of individuals and social groups: motivations to satisfy individual biological needs, motivations to coordinate social interactions, and motivations to ensure the survival and maintenance of group interests. Based on different types of motivations, the initial theoretical model proposed by Schwartz (1992) comprised only 10 basic individual values; however, as research advanced, Schwartz et al. (2012) refined these 10 basic individual values and supplemented additional types of basic individual values, ultimately expanding to 19 basic individual values.

**FIGURE 1:**
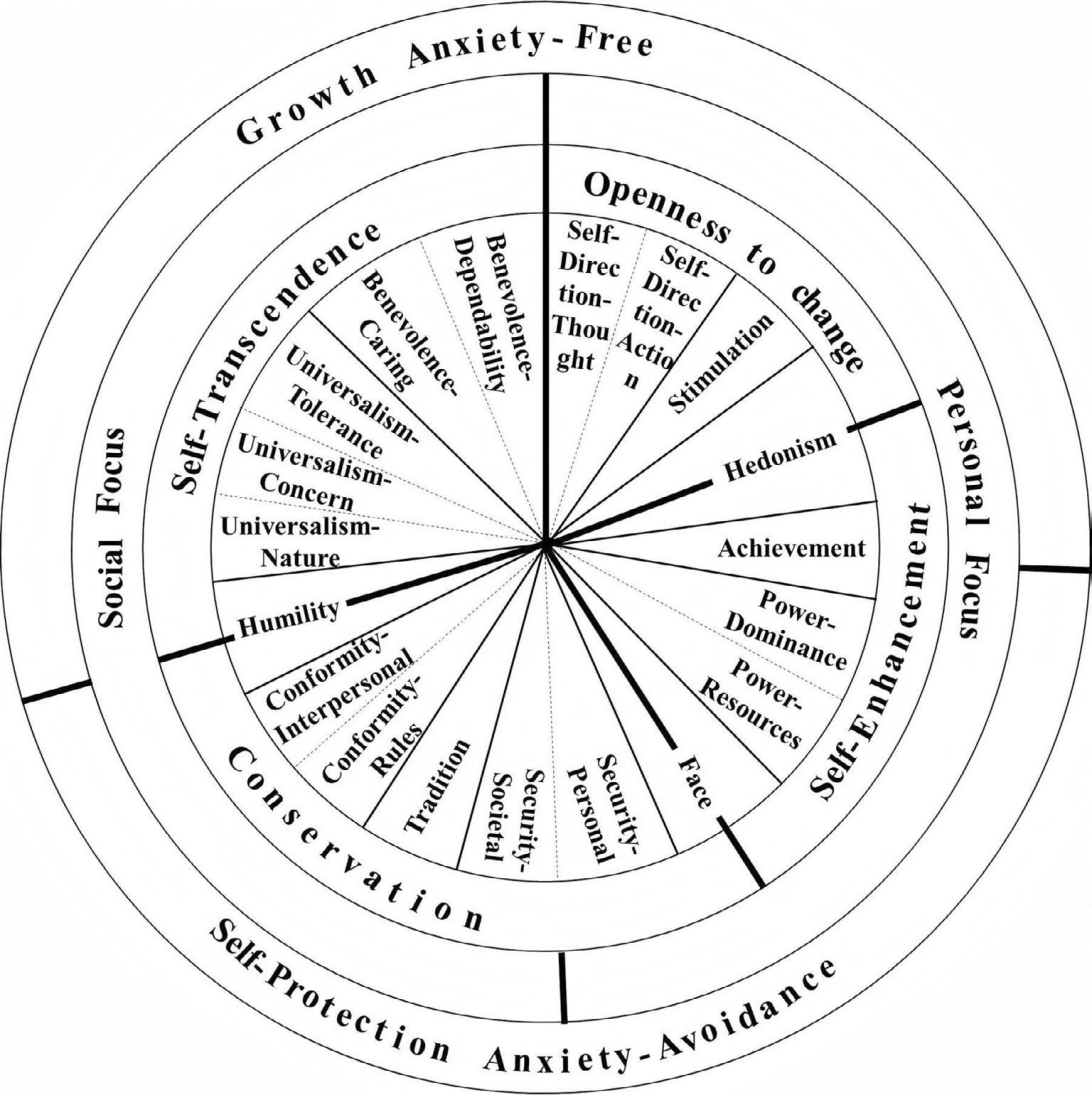
Circular structure of personal values (adapted from Schwartz and Cieciuch 2021).

Each basic individual value represents different motivations, and by systematically arranging basic individual values with similar motivations, a motivational continuum is formed (Figure 1) (Schwartz 1992, 2006; Schwartz et al. 2012). There is no intrinsic similarity or conflict among different individual values, but rather, it is essentially determined by the internal motivations they embody. The distance between individual values reflects the similarity of their internal motivations. Specifically, the closer two individual values are, the more similar their internal motivations are; such as achievement (AC) and hedonism (HE) both prioritize self‐gratification; the farther two individual values are, the more conflicting their internal motivations are; such as Self‐Direction‐Thought and Self‐Direction‐Action underscores individual pursuit of self‐expression and independence, while Tradition (TR) values respect and adherence to traditional norms and societal established practices.

Furthermore, building upon the internal motivations of adjacent individual values, Schwartz et al. (2012) delineated four pairs of higher‐order values on top of the 19 basic individual values: (1) openness to change (OP) versus conservation (CON), where the former actively promotes innovation, adventure, and freedom, while the latter upholds stability, security, and TR; (2) self‐enhancement (SEN) versus self‐transcendence (STR), where the former prioritizes personal success and power, while the latter underscores transcending self‐interest and caring for the welfare of others and the environment; (3) personal focus versus social focus, where the former emphasizes individual needs and desires, while the latter concentrates on collective and societal interests; (4) growth anxiety‐free versus self‐protection anxiety‐avoidance, where the former facilitates personal growth and self‐actualization, mainly based on internal motivations, while the latter focuses on avoiding threats and self‐protection, mainly based on external motivations.

Every individual's value system encompasses 19 basic individual values and 8 higher‐order values. However, these values demonstrate differential prioritization within personal value systems, with different individuals exhibiting divergent valuation patterns toward the same value. For instance, some may consider AC as a central tenet compared to other values, while others might assign primacy to TR. Consequently, individuals develop their value hierarchies based on the relative ranking of values (Schwartz 2012). This hierarchical structure subsequently orchestrates daily behaviors—the greater the importance of a value to an individual, the stronger its behavioral determinacy (Schwartz 2012). For example, if someone strongly prioritizes stimulation (ST) and HE, their behaviors will predominantly align with self‐stimulation orientation, while other values exert comparatively diminished influence.

### 1.2 The Current Study

From the perspective of traditional culture, Chinese people have been profoundly shaped by Confucianism, which emphasizes social stability and order, with core values including “filial piety,” “collectivism,” and “social harmony.” These cultural values have cultivated strong family‐nation sentiments among Chinese people (Zhong and Li 2025). Regarding contemporary national development, the Chinese government has proposed the Core Socialist Values for domestic society (The State Council of the People's Republic of China, 2013) and the concept of “a Community with a Shared Future for Mankind” on the global stage (Shao 2017). These initiatives reflect Chinese culture's emphasis on CON and STR values (Wang et al. 2025), which stand in sharp contrast to Western individualism (Jiang 2016). As demonstrated in prior research (Jin et al. 2019), the principles most valued by Chinese people in descending priority are conformity‐rules (COR), benevolence‐dependability (BED), benevolence‐caring (BEC), security‐societal (SES), and security‐personal (SEP), whereas the least valued principles in ascending order are power‐resources (POR), power‐dominance (POD), TR, ST, and face (FAC).

In fact, some researchers have already explored the applicability of this model within Chinese cultural contexts (Heim et al. 2018; Jin et al. 2019; Schwartz and Cieciuch 2021). However, these studies have some limitations. Heim et al. (2018) conducted a reanalysis of Chinese data from a previous meta‐analysis (Steinmetz et al. 2012), revealing that five out of six samples demonstrated relatively poor fit with the theoretical structure. Subsequently, they implemented a large‐scale study involving 10,652 Chinese university students, and the data confirmed the applicability of the circular structure of personal values in this cultural setting. They speculated that the small sample size in Study 1 might account for the incomplete alignment with the circular structure. Nevertheless, the participants in this study were exclusively recruited from four undergraduate institutions in China, and the original 10‐factor model was tested using the abbreviated version of the PVQ‐21 questionnaire.

Jin et al. (2019) validated the 19‐factor model of values by revising the Portrait Value Questionnaire (Schwartz et al. 2012). They collected 2569 valid responses from diverse populations through multiple channels. The results demonstrated satisfactory structural validity and reliability of the model, though discrepancies emerged between the original model and the interpretation of the value structure among Chinese participants. For instance, AC values exhibited significant associations with geographically distal values but weaker correlations with proximal ones. However, this study determined the positions of the 19 personal values solely through intra‐correlation coefficients, failing to capture their circular structure and spatial configuration. Moreover, the measurement instrument employed in this study was somewhat outdated. The PVQ for assessing the 19 personal values has undergone several iterations since 2012 (PVQ‐5X → PVQ‐R → PVQ‐RR; Schwartz and Cieciuch 2021).

Schwartz and Cieciuch (2021) employed the PVQ‐RR to assess the reliability, structural validity, circular structure, and cross‐cultural measurement invariance of the 19 personal values in 49 cultural groups worldwide (totaling 53,472 participants). The results indicated that the PVQ‐RR demonstrated reliable measurement of the 19 personal values in the majority of cultural groups; multidimensional scaling (MDS) analyses validated the theoretical assumption of the 19 personal values being organized around a circle in diverse cultural contexts. However, given that the purpose of this study was to validate the reliability of the PVQ‐RR and the 19‐factor model in cross‐cultural comparisons, the results pertaining to the Chinese group were not elaborated in depth. Moreover, the representativeness of the Chinese participants included (*n* = 1201; data collection period: 2017–2020) could not be systematically ascertained.

In addition to the aforementioned limitations of insufficient sample representativeness, outdated measurement tools, and methodological incompleteness, we must also account for the influence of societal environment on personal values. Notably, the data collection periods in all three cited studies occurred prior to 2020—that is, preceding the global outbreak of the COVID‐19 pandemic. This pandemic profoundly altered human lifestyles and social structures, and such a cataclysmic global event would inevitably exert far‐reaching effects on personal values (Sagiv and Schwartz 2021). For instance, the health crises and economic pressures triggered by the pandemic may heighten individuals' prioritization of fundamental needs like health, safety, and financial stability (Fenner and Cernev 2021), potentially elevating the relative significance of security values.

In summary, to validate the applicability of the Theory of Individual Values and the PVQ‐RR in the Chinese cultural context and to address the limitations of existing research, it is necessary to conduct new data collection for exploration. Furthermore, we incorporated gender and age group analysis. Prevailing social norms for men and women may result in different value priorities (Sagiv et al. 2017). Moreover, the social environment evolves with historical progression, which may lead to variations in the applicability of PVQ‐RR across different age groups. To assess the reliability and validity of the Chinese version of PVQ‐RR in the Chinese population, this study categorized the subjects into different age cohorts with a 10‐year interval. However, since adolescence represents a pivotal period for the formation and development of personal values, we stratified adolescents aged 11–20 into early adolescence (11–13 years old), middle adolescence (14–17 years old), and late adolescence (18–20 years old) according to previous research (Smetana et al. 2006).

## 2 Methods

### 2.1 Participants

This study utilized simple random sampling from June 2023 to May 2024, collecting a total of 9590 valid questionnaires through both paper‐based and online surveys after excluding responses that failed the lie detection items. The ages ranged from 11 to 60 years (*M* = 20.72, SD = 10.03), with 312 missing age data; 4360 males (45.5%), 4436 females (46.3%), and 794 with unreported gender data (see Table 1 for details).

**TABLE 1:** Distribution of people in different age stages.

Age	Male	Female	Total
11–13	876	885	1761
14–17	1882	1323	3205
18–20	501	516	1017
21–30	615	610	1225
31–40	96	481	577
41–50	211	428	639
51–60	48	43	91
Total	4229	4286	8515

The online questionnaires targeted adult participants, who received personalized values assessment reports upon completion. Paper questionnaires were administered to minors (secondary school students) with written informed consent obtained from the students themselves, their guardians, and homeroom teachers prior to administration. Homeroom teachers facilitated the paper questionnaires to their classes, with students completing them during scheduled class time. Participating schools subsequently received aggregate values assessment reports for their student populations.

The number of cases with missing item data ranged from 8 to 71, accounting for 0.1%–0.7% of responses; the number of missing items per case ranged from 1 to 6, representing 1.8%–10.5% of items. Using Little's MCAR test to assess the type of missing data, the results showed *χ* ^2^ = 29565.22, df = 22,238, *p <* 0.001, indicating that the data were not Missing Completely at Random. Therefore, we employed Full Information Maximum Likelihood for data processing.

### 2.2 Measures

The PVQ‐RR: This questionnaire was developed by Schwartz and Cieciuch (2021) to measure 19 basic personal values, with 3 items under each personal value, totaling 57 items. The questionnaire uses a 6‐point scale: 1 = *not like me at all*, 2 = *not like me*, 3 = *kind of like me*, 4 = *somewhat like me*, 5 = *like me*, and 6 = *like me very much*. In the study by Schwartz and Cieciuch (2021), the Cronbach's *α* of the 19 value types in the Chinese sample ranged from 0.43 to 0.83. The fit indices of the four higher‐order values were good.

The Chinese‐adapted PVQ‐RR used in this study was developed by the authors through the following rigorous procedures: (1) The authors requested the original PVQ‐RR from Prof. Schwartz via email; (2) doctoral‐level English translation specialists translated the questionnaire into Chinese; (3) Chinese linguistics experts at the doctoral level refined the linguistic expressions in the initial translation; (4) two senior psychology professors evaluated the refined Chinese version; (5) an English language specialist conducted back‐translation comparison with the original to verify conceptual equivalence; (6) professional translators reversed‐translated the Chinese version into English, with both versions submitted to Prof. Schwartz for review; (7) the finalized Chinese version received official approval from Prof. Schwartz. All personnel involved in Steps 2–6 worked independently, with communication exclusively mediated by the authors.

The adapted Chinese version retained all original dimensions, response format, item count, and substantive content without modification.

### 2.3 Procedure

1. Assess the reliability of 19 basic personal values and 4 higher‐order values for the total sample and subsamples (gender and age groups) by computing Cronbach's *α* coefficient.
2. Evaluate the validity of the 4 higher‐order values for the total sample and subsamples (gender and age groups) through confirmatory factor analysis (CFA). A model demonstrates good validity when the comparative fit index (CFI) and Tucker–Lewis index (TLI) are both ≥ 0.90, and the standardized root mean square residual (SRMR) and root mean square error of approximation (RMSEA) are both ≤ 0.80. An acceptable model requires CFI and TLI > 0.80, and SRMR and RMSEA < 0.10 (Wang and Bi 2018).
3. Conduct measurement invariance analyses with multigroup CFA (MGCFA) to test the consistency of the four higher‐order values across gender and age groups. A nonsignificant difference between models is indicated if ΔCFI and ΔTLI < 0.01; moderate differences if 0.01–0.02; and substantial differences if > 0.02 (Wang and Bi 2018).
4. First, derive the two‐dimensional spatial coordinates and circular structure of the total sample and subsamples (gender and age groups) via MDS; subsequently, employ Procrustes rotation to compare each subsample against the total sample's coordinates. Consistency is quantified using Tucker's phi coefficient (> 0.90 = high consistency; < 0.85 = inconsistency; ten Berge 1986).
5. Determine the relative importance of personal values for the total sample and subsamples (gender and age groups). Each item's score was centered by subtracting the grand mean of all items, followed by computing type‐specific mean scores for ranking the 19 values in descending order. Higher scores reflect greater value importance.

Analytical tools: Steps 1, 4, and 5 were performed in SPSS 25.0; Steps 2 and 3 were executed in Mplus 8.3.

## 3 Results

### 3.1 Reliability

Table 2 presents the Cronbach's *α* coefficients for the 19 basic personal values across the total sample and subsamples (gender and age groups). The reliability coefficients for the 19 personal values in the total sample span from 0.47 to 0.81 (*M* = 0.69, SD = 0.09). The average Cronbach's *α* for the 19 personal values across all subsamples varies between 0.65 and 0.73.

**TABLE 2:** Cronbach's *α* coefficient for 19 basic personal values.

	HUM	UNN	UNC	UNT	BEC	BED	SDT	SDA	ST	HE	AC	POD	POR	FAC	SEP	SES	TR	COR	COI
Whole sample	0.47	0.81	0.75	0.69	0.74	0.55	0.73	0.71	0.62	0.74	0.68	0.72	0.78	0.54	0.68	0.80	0.68	0.73	0.71
Male	0.46	0.81	0.75	0.69	0.73	0.55	0.71	0.70	0.59	0.72	0.69	0.73	0.78	0.54	0.66	0.79	0.67	0.74	0.70
Female	0.48	0.80	0.75	0.69	0.75	0.55	0.73	0.72	0.63	0.76	0.69	0.71	0.78	0.54	0.70	0.81	0.66	0.72	0.72
Age 11–13	0.44	0.76	0.72	0.68	0.7	0.52	0.66	0.66	0.53	0.71	0.65	0.63	0.78	0.49	0.65	0.75	0.66	0.72	0.71
Age 14–17	0.46	0.84	0.74	0.69	0.74	0.54	0.75	0.71	0.59	0.74	0.67	0.66	0.78	0.51	0.69	0.83	0.66	0.74	0.73
Age 18–20	0.47	0.77	0.74	0.64	0.67	0.54	0.69	0.64	0.59	0.66	0.66	0.75	0.71	0.52	0.57	0.75	0.64	0.67	0.73
Age 21–30	0.40	0.78	0.72	0.65	0.70	0.54	0.7	0.66	0.63	0.71	0.61	0.74	0.69	0.59	0.59	0.79	0.69	0.71	0.67
Age 31–40	0.48	0.81	0.76	0.72	0.79	0.64	0.74	0.76	0.67	0.74	0.73	0.75	0.76	0.62	0.78	0.80	0.71	0.78	0.64
Age 41–50	0.55	0.83	0.78	0.75	0.79	0.65	0.72	0.77	0.68	0.74	0.73	0.78	0.79	0.58	0.81	0.83	0.73	0.78	0.66
Age 51–60	0.51	0.86	0.79	0.75	0.71	0.61	0.69	0.76	0.72	0.73	0.78	0.74	0.78	0.52	0.78	0.83	0.76	0.76	0.70

Abbreviations: AC = achievement; BEC = benevolence‐caring; BED = benevolence‐dependability; COI = conformity‐interpersonal; COR = conformity‐rules; FAC = face; HE = hedonism; HUM = humility; POD = power‐dominance; POR = power‐resources; SDA = self‐direction‐action; SDT = self‐direction‐thought; SEP = security‐personal; SES = security‐societal; ST = stimulation; TR = tradition; UNC = universalism‐concern; UNN = universalism‐nature; UNT = universalism‐tolerance; the same as following tables.

Table 3 displays the Cronbach's *α* coefficients for the four higher‐order value types across the total sample and subsamples (gender and age groups). The reliability coefficients for the four higher‐order value types in the total sample span from 0.83 to 0.90 (*M* = 0.87, SD = 0.03). The average Cronbach's *α* for the 4 higher‐order value types across all subsamples varies between 0.84 and 0.91.

**TABLE 3:** Cronbach's *α* coefficient for four higher‐order values.

	Self‐transcendence (UNN, UNC, UNT, BEC, BED, HUM)	Openness to change (SDA, SDT, ST, HE)	Conservation (SEP, SES, COI, COR, FAC, TR)	Self‐enhancement (AC, POD, POR)
Whole sample	0.90	0.86	0.89	0.83
Male	0.90	0.85	0.89	0.84
Female	0.90	0.87	0.88	0.83
Age 11–13	0.88	0.82	0.87	0.80
Age 14–17	0.89	0.84	0.88	0.80
Age 18–20	0.88	0.83	0.86	0.82
Age 21–30	0.88	0.83	0.86	0.81
Age 31–40	0.93	0.88	0.91	0.85
Age 41–50	0.94	0.89	0.93	0.87
Age 51–60	0.94	0.89	0.92	0.84

### 3.2 Structural Validity

Table 4 presents the CFA fit indices for STR for the total sample and subsamples (gender and age groups). Only two samples demonstrate TLI > 0.85, while the CFI and TLI of all other samples exceed 0.90; the SRMR and RMSEA of all samples fall below 0.08.

**TABLE 4:** Fit indices of CFA for self‐transcendence.

	Chi^2^	df	CFI	TLI	SRMR	RMSEA	90% CI
Whole sample	4571.336	120	0.931	0.912	0.037	0.062	[0.061–0.064]
Male	2303.798	120	0.925	0.905	0.039	0.065	[0.062–0.067]
Female	2219.971	120	0.932	0.914	0.038	0.063	[0.061–0.065]
Age 11–13	806.899	120	0.933	0.915	0.035	0.056	[0.052–0.06]
Age 14–17	1377.988	120	0.942	0.926	0.034	0.056	[0.054–0.059]
Age 18–20	739.747	120	0.895	0.866	0.049	0.07	[0.065–0.075]
Age 21–30	902.95	120	0.915	0.892	0.043	0.063	[0.059–0.067]
Age 31–40	618.029	120	0.916	0.893	0.047	0.079	[0.073–0.085]
Age 41–50	654.707	120	0.924	0.904	0.044	0.08	[0.074–0.086]
Age 51–60	260.629	120	0.875	0.841	0.062	0.108	[0.09–0.126]

Abbreviations: CFI = comparative fit index; CI = confidence interval; df = degrees of freedom; RMSEA = root mean square error of approximation; SRMR = standardized root mean square residual; TLI = Tucker–Lewis index; the same as following tables.

Table 5 displays the CFA fit indices for OP for the total sample and subsamples (gender and age groups). The CFI and TLI of all samples are above 0.90; the SRMR and RMSEA of all samples are under 0.08.

**TABLE 5:** Fit indices of CFA for openness to change.

	Chi^2^	df	CFI	TLI	SRMR	RMSEA	90% CI
Whole sample	1529.518	48	0.958	0.943	0.03	0.057	[0.054–0.059]
Male	713.014	48	0.955	0.939	0.031	0.056	[0.053–0.06]
Female	772.061	48	0.959	0.944	0.03	0.058	[0.055–0.062]
Age 11–13	408.274	48	0.93	0.904	0.037	0.064	[0.059–0.07]
Age 14–17	614.193	48	0.951	0.933	0.036	0.06	[0.056–0.064]
Age 18–20	282.268	48	0.925	0.897	0.041	0.068	[0.061–0.076]
Age 21–30	315.31	48	0.95	0.931	0.036	0.058	[0.052–0.064]
Age 31–40	221.977	48	0.945	0.925	0.038	0.074	[0.064–0.084]
Age 41–50	168.075	48	0.964	0.951	0.033	0.06	[0.05–0.07]
Age 51–60	100.894	48	0.89	0.849	0.057	0.104	[0.076–0.133]

Table 6 illustrates the CFA fit indices for CON for the total sample and subsamples (gender and age groups). Only four samples exhibit CFI > 0.80, with the rest surpassing 0.90; only one sample achieves TLI > 0.90, with the rest maintaining > 0.80; only one sample shows RMSEA < 0.10, with the SRMR and RMSEA of all other samples below 0.08.

**TABLE 6:** Fit indices of CFA for conservation.

	Chi^2^	df	CFI	TLI	SRMR	RMSEA	90% CI
Whole sample	5573.96	120	0.908	0.883	0.054	0.069	[0.067–0.07]
Male	2300.337	120	0.918	0.896	0.049	0.065	[0.062–0.067]
Female	3193.834	120	0.891	0.861	0.06	0.076	[0.074–0.078]
Age 11–13	982.829	120	0.913	0.889	0.049	0.063	[0.059–0.067]
Age 14–17	2010.416	120	0.908	0.883	0.053	0.069	[0.067–0.072]
Age 18–20	752.938	120	0.885	0.853	0.067	0.071	[0.066–0.076]
Age 21–30	1050.746	120	0.896	0.867	0.065	0.069	[0.065–0.072]
Age 31–40	612.015	120	0.91	0.886	0.06	0.079	[0.072–0.085]
Age 41–50	641.897	120	0.921	0.899	0.06	0.079	[0.073–0.085]
Age 51–60	342.574	120	0.792	0.735	0.076	0.136	[0.119–0.152]

Table 7 summarizes the CFA fit indices for SEN for the total sample and subsamples (gender and age groups). Only four samples attain TLI > 0.85, while the CFI and TLI of all other samples are greater than 0.90; only four samples yield RMSEA < 0.10, with the SRMR and RMSEA of all other samples less than 0.08.

**TABLE 7:** Fit indices of CFA for self‐enhancement.

	Chi^2^	df	CFI	TLI	SRMR	RMSEA	90% CI
Whole sample	1870.604	24	0.93	0.895	0.044	0.09	[0.086–0.093]
Male	849.383	24	0.934	0.901	0.043	0.089	[0.084–0.094]
Female	974.432	24	0.922	0.883	0.045	0.094	[0.089–0.1]
Age 11–13	338.076	24	0.925	0.887	0.045	0.085	[0.077–0.093]
Age 14–17	544.484	24	0.935	0.903	0.047	0.081	[0.075–0.087]
Age 18–20	300.259	24	0.899	0.849	0.054	0.105	[0.094–0.116]
Age 21–30	371.481	24	0.912	0.867	0.049	0.094	[0.085–0.102]
Age 31–40	178.858	24	0.928	0.892	0.044	0.099	[0.085–0.112]
Age 41–50	170.472	24	0.945	0.918	0.041	0.093	[0.08–0.107]
Age 51–60	49.241	24	0.925	0.888	0.068	0.102	[0.061–0.143]

### 3.3 Measurement Invariance

The gender‐based measurement invariance results are presented in Table 8. Notably, the discrepancies in fit indices between the Configural Invariance, Metric Invariance, and Scalar Invariance models across the four higher‐order values by gender were all below 0.01.

**TABLE 8:** Fit indices of measurement invariance by gender.

	Chi^2^	df	CFI	TLI	SRMR	RMSEA	90% CI	ΔCFI	ΔTLI
Self‐transcendence									
Configural	4523.769	240	0.929	0.909	0.038	0.064	[0.062–0.065]		
Metric	4547.883	252	0.929	0.913	0.039	0.062	[0.061–0.064]	0	0.004
Scalar	4674.29	264	0.927	0.915	0.04	0.062	[0.06–0.063]	−0.002	0.002
Openness to change									
Configural	1485.076	96	0.957	0.941	0.031	0.057	[0.055–0.06]		
Metric	1490.023	104	0.957	0.946	0.031	0.055	[0.053–0.058]	0	0.005
Scalar	1526.198	112	0.957	0.949	0.031	0.054	[0.051–0.056]	0	0.003
Conservation									
Configural	5494.171	240	0.904	0.878	0.055	0.071	[0.069–0.072]		
Metric	5513.478	252	0.904	0.883	0.056	0.069	[0.067–0.07]	0	0.005
Scalar	5656.034	264	0.902	0.886	0.057	0.068	[0.067–0.07]	−0.002	0.003
Self‐enhancement									
Configural	1823.814	48	0.928	0.893	0.044	0.092	[0.088–0.095]		
Metric	1835.846	54	0.928	0.904	0.045	0.087	[0.083–0.09]	0	0.011
Scalar	1887.463	60	0.926	0.912	0.046	0.083	[0.08–0.086]	−0.002	0.008

The age‐group measurement invariance results are displayed in Table 9. Of particular interest, while the discrepancies in fit indices between the Configural Invariance and Metric Invariance models across the four higher‐order value types by age group were all below 0.01, those between the Metric Invariance and Scalar Invariance models consistently exceeded 0.02.

**TABLE 9:** Fit indices of measurement invariance by age group.

	Chi2	df	CFI	TLI	SRMR	RMSEA	90% CI	ΔCFI	ΔTLI
Self‐transcendence									
Configural	5360.95	840	0.926	0.906	0.04	0.064	[0.062–0.065]		
Metric	5586.312	912	0.924	0.911	0.048	0.062	[0.061–0.064]	−0.002	0.005
Scalar	7290.914	984	0.897	0.888	0.058	0.07	[0.068–0.071]	−0.027	−0.023
Openness to change									
Configural	2110.992	336	0.945	0.924	0.037	0.063	[0.061–0.066]		
Metric	2207.573	384	0.943	0.932	0.043	0.06	[0.057–0.062]	−0.002	0.008
Scalar	3054.108	432	0.919	0.913	0.053	0.068	[0.065–0.07]	−0.024	−0.019
Conservation									
Configural	6393.427	840	0.904	0.878	0.058	0.071	[0.069–0.072]		
Metric	6776.4	912	0.899	0.881	0.064	0.07	[0.068–0.071]	−0.005	0.003
Scalar	8235.633	984	0.875	0.864	0.072	0.075	[0.073–0.076]	−0.024	−0.017
Self‐enhancement									
Configural	1952.872	168	0.926	0.889	0.047	0.09	[0.086–0.093]		
Metric	2277.495	204	0.914	0.894	0.062	0.088	[0.084–0.091]	−0.012	0.005
Scalar	3092.338	240	0.882	0.876	0.07	0.095	[0.092–0.098]	−0.032	−0.018

### 3.4 Circular Structure of Values

**FIGURE 2:**
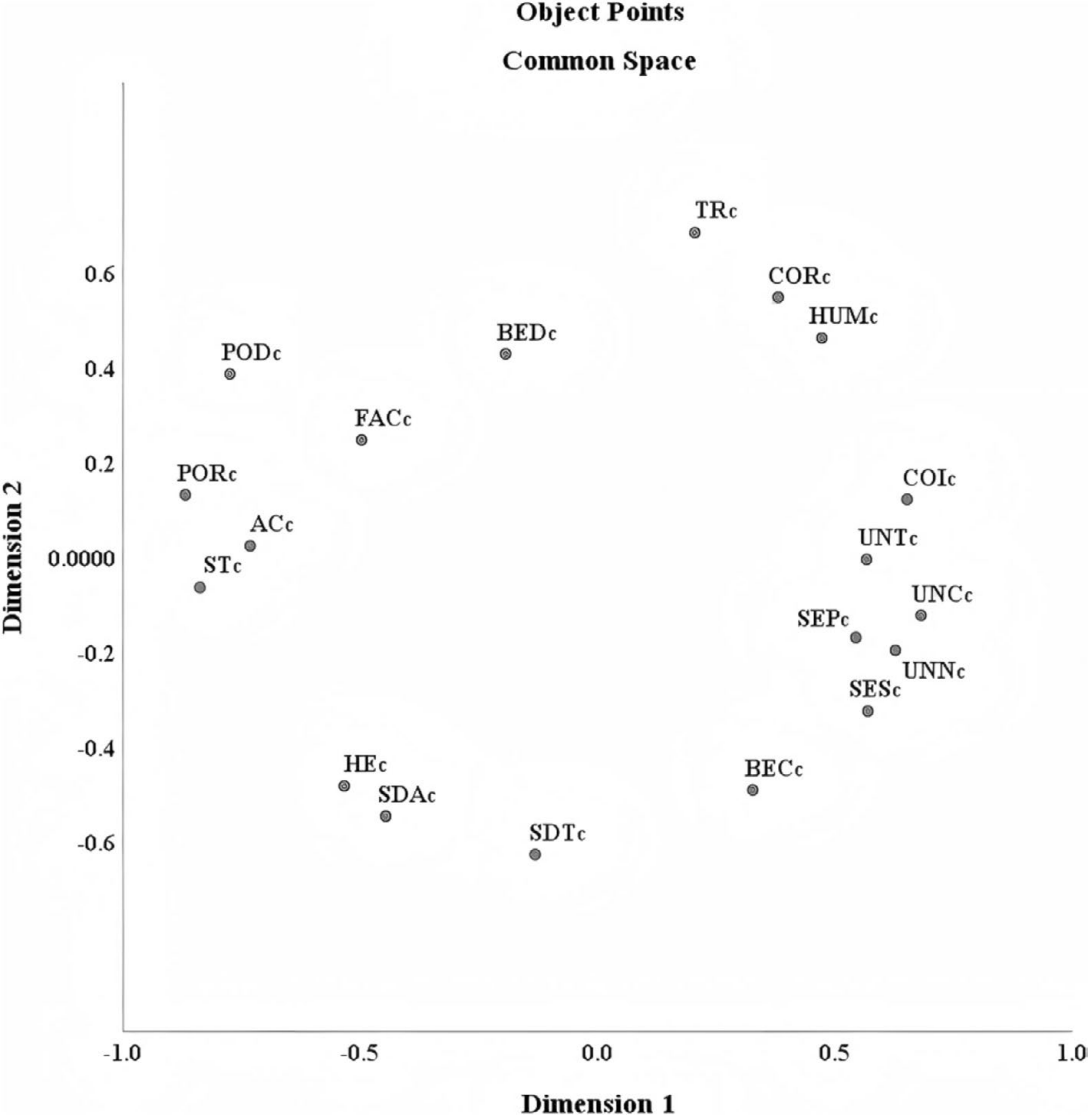
MDS two‐dimensional projection of 19 centered values based on correlation matrix. *Note*: The c following each label indicates that the value is centered.

We employed MDS to map the 19 basic personal values into a two‐dimensional space after centralization (see Figure 2). Overall, the positions of the four higher‐order values in this space exhibited a rotation compared to the theoretical model. STR occupies the lower right, OP the lower left, CON the upper right, and SEN the upper left. However, BED is situated between SEN and CON; SEP and SES are proximal to STR, with a spatial position nearer to Universalism.

Next, using these as target coordinates, we assessed the coordinates of each subsample (gender and age groups) against the target coordinates, as shown in Table 10. Specifically, in the first dimension, excluding the samples aged 31–40 (< 0.85) and 51–60 (< 0.80), the Tucker's phi coefficient for all other subsamples exceeded 0.90; in the second dimension, excluding the samples aged 41–50 (< 0.90) and 51–60 (< 0.80), the Tucker's phi coefficient for all other subsamples was above 0.90. Supporting Information provides the two‐dimensional coordinates of the total sample and subsamples.

**TABLE 10:** Tucker's phi coefficient.

	Dimension1	Dimension 2
Male	0.9952	0.9877
Female	0.9965	0.9834
Age 11–13	0.9895	0.9699
Age 14–17	0.9907	0.9651
Age 18–20	0.9752	0.9426
Age 21–30	0.9295	0.9337
Age 31–40	0.8475	0.9247
Age 41–50	0.9280	0.8887
Age 51–60	0.7833	0.4764

### 3.5 Relative Importance

Table 11 presents the rankings of the 19 personal values based on their relative significance for the total sample and subsamples (gender and age groups). Except for the age 31–40 group, SES consistently occupies the top position in terms of importance across the overall sample and other subsamples; POR and POD invariably rank lowest in all samples.

**TABLE 11:** Relative importance ranking of 19 basic personal values across all samples.

Rank	Whole sample	Male	Female	Age 11–13	Age 14–17	Age 18–20	Age 21–30	Age 31–40	Age 41–50	Age 51–60
1	SES	SES	SES	SES	SES	SES	SES	SEP	SES	SES
2	BEC	BEC	UNN	SEP	BEC	BEC	BEC	SES	SEP	SEP
3	UNN	SEP	SEP	UNN	UNN	SDA	SDA	UNN	COI	UNC
4	SEP	UNN	BEC	BEC	SEP	UNN	UNN	COI	UNC	UNN
5	SDA	SDA	UNC	UNC	SDA	HE	SEP	BEC	UNN	COI
6	UNC	UNC	SDA	COI	HE	SDT	SDT	UNC	BEC	BEC
7	HE	COI	COI	SDA	UNC	SEP	HE	SDA	SDA	UNT
8	COI	HE	HE	HE	COI	UNC	UNC	UNT	UNT	SDA
9	SDT	SDT	SDT	SDT	SDT	COI	UNT	SDT	SDT	SDT
10	UNT	UNT	UNT	UNT	UNT	UNT	FAC	FAC	BED	FAC
11	FAC	BED	FAC	AC	FAC	FAC	COI	HE	FAC	BED
12	BED	FAC	BED	FAC	BED	BED	AC	BED	TR	HE
13	AC	AC	AC	BED	AC	AC	BED	AC	HE	TR
14	COR	COR	HUM	COR	COR	HUM	HUM	TR	AC	AC
15	HUM	HUM	TR	HUM	HUM	ST	ST	HUM	HUM	COR
16	TR	TR	COR	TR	TR	TR	COR	COR	COR	HUM
17	ST	ST	ST	ST	ST	COR	TR	ST	ST	ST
18	POR	POR	POR	POR	POR	POR	POR	POR	POR	POR
19	POD	POD	POD	POD	POD	POD	POD	POD	POD	POD

Men and women exhibit identical rankings for SES, SEP, conformity‐interpersonal (COI), HE, self‐direction‐thought (SDT), universalism‐tolerance (UNT), AC, ST, POR, and POD; men prioritize BEC, self‐direction‐action (SDA), BED, and COR more highly, whereas women assign greater importance to universalism‐nature (UNN), universalism‐concern (UNC), FAC, humility (HUM), and TR.

## 4 Discussion

This study administered psychometric tests on the Chinese version of PVQ‐RR using a large sample with an age range from 11 to 60 to examine the applicability of the Theory of Individual Values in the Chinese context. The results demonstrate that the Chinese version of PVQ‐RR is suitable for both genders and across age groups, further corroborating the cross‐cultural consistency of the Theory of Individual Values.

### 4.1 Reliability and Structural Validity

First, the internal consistency coefficients of the 19 basic personal values across all samples were at a relatively low level. This aligns with prior findings, as each personal value is assessed by merely three items (Schwartz and Cieciuch 2021). Nevertheless, this does not compromise the validity of our measurement for the 19 values. Notably, the Cronbach's *α* coefficient for HUM consistently ranked the lowest and remained below 0.50 (except for the age 41–50 and age 51–60 groups). This observation is corroborated by earlier studies (Jin et al. 2019; Schwartz and Cieciuch 2021) and is evident across diverse cultural groups (Schwartz and Cieciuch 2021). Schwartz and Cieciuch (2021) posited that HUM spans both CON and STR dimensions, and its intrinsic meaning is challenging to capture with only three items, thereby yielding this outcome. We deem this explanation plausible, given that fewer items inherently limit the ability to encapsulate the true essence of a complex construct (Reise et al. 2000).

Second, the Cronbach's *α* coefficients for the four higher‐order values exceeded 0.80 in all samples. Although the CFA results for CON were moderately weaker compared to STR, OP, and SEN, they still fell within an acceptable range. These findings are also consistent with previous research (Schwartz and Cieciuch 2021), indicating that the Chinese version of PVQ‐RR reliably measures the 4 higher‐order values among Chinese populations.

### 4.2 Measurement Invariance

This study performed the measurement invariance test of the four higher‐order values by gender and age group in the Chinese cultural context for the first time. The results demonstrate that the conceptualizations of STR, OP, CON, and SEN are highly consistent across different genders and age groups, thereby reinforcing the validity of the Chinese version of PVQ‐RR.

The measurement invariance results by gender reveal that the four higher‐order values are equivalent in construct form, measurement, and scale. Specifically, between gender groups, the items measuring the four higher‐order values share the same units and reference points, and the latent variable scores derived from the observed variables are unbiased; consequently, researchers can reliably compare the value scores of different gender groups, verifying that these differences reflect true group differences rather than measurement artifacts (He and Li 2020).

The measurement invariance results by age group indicate that the four higher‐order values are equivalent in structural form and measurement parameters but not in scale. This finding means that the factor structure and measurement relationships of the four higher‐order values are invariant across age groups. However, different age groups may exhibit different response styles or baseline levels when evaluating these value questions. We hypothesize that societal evolution has engendered value differences among different age groups. The remarkable development of China in recent decades is undeniable, and the varying stages of national development shape the sociohistorical context and cultural transformations, ultimately leading to differences in collective ideologies, attitudes, and behaviors (Chen and Lian 2011; Wang and You 2013).

### 4.3 Circular Structure of Values

The circular structure of Chinese personal values not only aligns with Schwartz's Theory of Individual Values but also reflects the distinctive value structure characteristics of Chinese people.

In the overarching structure, the results of this study support the theoretical model, where STR is opposite to SEN, and OP is opposite to CON; these two pairs of higher‐order values located in opposite positions intersect each other. This finding is in line with prior research (Heim et al. 2018; Schwartz and Cieciuch 2021), demonstrating that the theory is not merely applicable to Western cultures but also accurately captures personal values in the Chinese cultural context, further validating its broad applicability.

Meanwhile, the positions of certain specific values diverge from those in the theoretical model, highlighting the unique value concepts of Chinese people. BED should be categorized under STR values like BEC, but in this study, it is located between SEN and CON, opposing BEC. This aligns with the MDS results of Schwartz and Cieciuch (2021), and the findings of Jin et al. (2019) also demonstrate a high positive correlation between AC and BED. This suggests that the BED value of Chinese people spans both SEN and CON. The underlying motivation of BED values is to become a reliable and trustworthy person (Schwartz et al. 2012). On the one hand, it reflects the virtue of “integrity”; only a person with integrity can earn others' trust. Chinese people prioritize integrity, as evidenced by numerous proverbs and stories on the subject (e.g., “a promise worth a thousand gold”); moreover, “trust” is a cornerstone of Confucian culture (Hou 2017). If a person seeks success or power, they must embody honesty and integrity; conversely, a person without integrity will fail to secure others' trust or attain significant accomplishments. On the other hand, it underscores a sense of responsibility. In family, work, and societal contexts, despite varying roles and responsibilities, Chinese people's expectations for each role converge on fulfilling one's duties (Lu and Gilmour 2004; Zhang 2015). Thus, becoming a reliable and trustworthy person equates to adhering to established social norms and traditional behavior patterns.

Furthermore, the SEP and SES values of Chinese people may demonstrate a stronger propensity to belong to STR. This constitutes a distinctive finding of this study. China is characterized by a profoundly collectivist culture (Hofstede 1993). In the Chinese cultural context, personal security is inextricably intertwined with collective security, and individual safety and interests are frequently embedded within the welfare of the collective (Gao 2020). By focusing on personal and social security, people are essentially attending to the well‐being of society at large. This resonates strongly with the STR values emphasizing concern for others and societal welfare.

After comparing the MDS coordinates of the gender and age subsamples with the overall coordinates, it was revealed that solely the 51–60 age group exhibited discrepancies with the overall structure in both dimensions. This divergence might stem from a substantial disparity in sample size compared to other groups or their formative experiences during childhood and adolescence. This cohort witnessed two pivotal historical events following the establishment of New China: the Cultural Revolution (1966–1976) and the Reform and Opening‐up (1978). Empirical evidence indicates that children as young as 5 years old possess the capacity to develop and formulate their own value structures (Döring et al. 2015; Lee et al. 2017).

### 4.4 Relative Importance

The personal value priorities of Chinese people also exhibit culturally distinctive characteristics compared to other cultures.

The most valued personal value for Chinese people is SES. This is not only inconsistent with findings from other cultures (where the top value is benevolence) (Schwartz 2012) but also diverges from the results of Jin et al. (2019) (where the top value was COR, and SES ranked fourth). This reflects the profound patriotic spirit of the Chinese people (Chen 2019) and underscores the high importance placed on social stability and order within Chinese culture. People aspire to live in a harmonious and orderly society. Moreover, the various measures taken by the state to protect people's lives during the COVID‐19 pandemic significantly strengthened national identity (Gong and Ye 2021).

BEC is the second‐highest value for Chinese people, reflecting the collectivist consciousness in Chinese culture, which emphasizes care and mutual assistance among people. The UNN value ranks third, indicating the growing importance that Chinese people place on environmental protection. This value is deeply rooted in traditional culture, such as the Daoist philosophy of “harmony between man and nature,” advocating for harmonious coexistence with nature. SEP ranks fourth, demonstrating the importance Chinese people attach to stability and security in personal life. Chinese culture emphasizes “settling down and enjoying work,” where personal security encompasses not only physical safety but also property security, psychological safety, and the stability of social status. SDA ranks fifth; although traditional Chinese culture emphasizes collectivism, with the modernization of society and the development of the economy and education, individual autonomy in behavioral choices is increasingly being valued (Schwartz and Cieciuch 2021). UNC ranks sixth, reflecting the respect Chinese people have for the rights of all people, underscoring equality and inclusiveness across social strata. HE ranks seventh in Chinese culture. The rise of this value reflects the shift in lifestyle perspectives among Chinese people following improvements in material conditions, prioritizing happiness and enjoyment in life, and pursuing a richer and more diverse life experience. COI ranks eighth, reflecting the focus on social order and interpersonal relationships in Chinese culture. Chinese society emphasizes harmony and order, and following rules is regarded as the foundation for maintaining social harmony. SDT ranks ninth, indicating that individual autonomy in thought may be partially constrained under the traditional collectivist cultural background.

UNT ranks 10th, positioned below UNN and UNC. This suggests that tolerance is not the foremost priority for individuals when encountering diverse cultures, beliefs, and lifestyles. FAC ranks 11th. In Chinese culture, “face” (mianzi) is a pivotal concept encompassing personal dignity, honor, and social status (Zhai 2004). However, contemporary priorities may deprioritize FAC as a central consideration in daily life. BED ranks 12th, reflecting a relatively diminished emphasis on the trustworthiness and reliability of others. AC ranks 13th; although success is a widely pursued objective, AC per se is not a dominant value in Chinese culture, where collective success and societal contribution hold greater weight. COR ranks 14th, demonstrating that while individuals adhere to fundamental social norms, this compliance is not a primary concern in their daily lives, as they may prioritize personal safety and freedom. HUM ranks 15th; despite HUM being regarded as a virtue in traditional Chinese culture, the rising significance of self‐expression has led to its reduced prominence as a personally emphasized trait. Nonetheless, HUM remains a respected quality. TR ranks 16th, highlighting a weaker emphasis on preserving and transmitting cultural, familial, and religious TRs, with individuals inclined toward personal freedom and social security rather than strict TR adherence. ST, POR, and POD, consistent with other cultures (Schwartz 2012), rank last. This indicates that Chinese people, akin to others, do not prioritize pursuing adventure, change, or control over others/material resources.

Regarding gender differences, as documented in prior research, the value priorities exhibit substantial consistency across genders, with only marginal variations (Costa et al. 2001; Döring et al. 2015). This study revealed that men assign greater importance to BEC, SDA, BED, and COR than women, whereas women prioritize UNN, UNC, FAC, HUM, and TR more significantly. Moreover, due to the lack of scalar measurement invariance across age groups, direct comparisons of value priorities between different age cohorts are precluded; nevertheless, their patterns align with the overall sample in ranking SES highest (except for the 31–40 age group) and evaluating POR and POD as least important. The observed preference for SEP over SES among individuals aged 31–40 may stem from their identity as the “new generation” emerging post China's Reform and Opening‐up policy. Their value development was deeply shaped by the sociohistorical context of that period: While China's accelerated integration with global systems fostered the adoption of Western individualism, the implementation of a market economy simultaneously amplified personal competitiveness, thereby redirecting the focus of security needs from collective welfare to individual capability attainment.

### 4.5 Implications

This study holds substantial theoretical and practical implications. Theoretically, first, it corroborates the applicability of Schwartz's Theory of Basic Human Values in non‐Western cultures (particularly China's collectivist context), thereby enhancing the theory's global generalizability. Second, the measurement invariance of the Chinese PVQ‐RR across genders and partial invariance across age groups establishes methodological foundations for cross‐group comparisons. Direct gender comparisons are methodologically sound, but age group differences require nuanced interpretation. Finally, the finding that BED in the Chinese sample demonstrates dual loading on both SEN and CON suggests potential need for culture‐specific refinements to Schwartz's theoretical model, particularly within Chinese cultural contexts.

Practically, first, the highest priority given to SES mirrors Chinese people's pronounced need for stable social order, suggesting policy prioritization toward public security systems (e.g., disaster response, cybersecurity) and social welfare provisions (e.g., healthcare, elderly care). Second, educational institutions can leverage the value priority findings to design culturally tailored curricula for Chinese adolescents, emphasizing national identity, collective consciousness, and social/environmental responsibilities. Third, corporations could utilize PVQ‐RR to assess employees' value orientations for strategic job placement—for instance, assigning BEC‐oriented individuals to collaborative roles and SDA‐oriented individuals to pioneering initiatives.

### 4.6 Limitations and Future Research

Despite the noteworthy insights provided by this study, there are still some limitations that need to be acknowledged. First, although the sample size is large and diverse in age, there is a marked difference in the sample size across different age groups, which may partially account for the significant differences in age group analysis in the study. Future research could replicate the results of this study while ensuring comparable sample sizes across age groups.

Second, the internal consistency coefficients of some basic personal values, particularly HUM, are suboptimal. This may stem from the limited number of items used to measure each value. Although this limitation is aligned with previous research, future studies could explore strategies to improve the reliability of these scales, such as incorporating additional items or refining existing ones to better reflect the complexity of certain values like HUM.

Furthermore, although the study confirmed measurement invariance between genders, the results revealed a lack of scalar invariance across different age groups. This suggests that response tendencies may differ with age, potentially influenced by generational differences or societal development in China. Future research could investigate these age‐related differences more thoroughly, possibly through longitudinal studies to examine how value systems evolve alongside changing social, political, and economic conditions.

Finally, while this study offers valuable insights into the cultural characteristics of values in China, subsequent research could explore the impact of additional demographic factors, such as socioeconomic status or educational attainment, on value priorities. Investigating these variables could yield a richer understanding of how diverse life experiences shape personal values among the Chinese population.

## 5 Conclusion

This study, through the examination of reliability, structural validity, MDS, and measurement invariance, confirmed that the Chinese version of PVQ‐RR can effectively assess the 19 basic personal values and four higher‐order values of Chinese people; this demonstrates that the Theory of Individual Values exhibits strong applicability in the Chinese cultural context. Therefore, future research may employ the Chinese version of PVQ‐RR to explore the potential relationships between Chinese people's values and other variables. This study also revealed that the most prioritized value for Chinese people is SES, which differs from other cultures; however, the least prioritized values—ST, POR, and POD—align with other cultural groups. This suggests that the value structure of Chinese people is culturally distinct. The value priorities of men and women are largely congruent for most values, with only minimal differences.

## Ethics Statement

All procedures performed in studies involving human participants followed the institutional and/or national research committee's ethical standards and the 1964 Helsinki declaration and its later amendments or comparable ethical standards. The study was approved by the Ethics Committee of Faculty of Psychology, Southwest University (IRB NO. H24067).

## Conflicts of Interest

The authors declare no conflicts of interest.

## Supporting information


**Data S1.** Supporting Information.

## Data Availability

The data that support the findings of this study are available on request from the corresponding author. The data are not publicly available due to privacy or ethical restrictions.

## References

[pchj70023-bib-0001] Chen, J. , and R. Lian . 2011. “A Review of the Development of Generational Work Value.” Advances in Psychological Science 19, no. 11: 1692–1701.

[pchj70023-bib-0002] Chen, L. 2019. “Lun Zhonghuaminzu Aiguozhuyi de Jingshen [On the Chinese Nation's Spirit of Patriotism].” Zhexue Yanjiu [Philosophical Research] 2019, no. 10: 11–19.

[pchj70023-bib-0003] Costa, P. , A. Terracciano , and R. McCrae . 2001. “Gender Differences in Personality Traits Across Cultures: Robust and Surprising Findings.” Journal of Personality and Social Psychology 81: 322–331. 10.1037//0022-3514.81.2.322. 11519935

[pchj70023-bib-0004] Döring, A. K. , S. H. Schwartz , J. Cieciuch , et al. 2015. “Cross‐Cultural Evidence of Value Structures and Priorities in Childhood.” British Journal of Psychology 106, no. 4: 675–699. 10.1111/bjop.12116. 25581067

[pchj70023-bib-0005] Fenner, R. , and T. Cernev . 2021. “The Implications of the Covid‐19 Pandemic for Delivering the Sustainable Development Goals.” Futures 128: 102726. 10.1016/j.futures.2021.102726. 34658398 PMC8510889

[pchj70023-bib-0006] Gao, L. 2020. “Family in China: The Characteristics of Chinese Culture in View of Liang Shu‐Ming.” Journal of East China Normal University(Humanities and Social Sciences) 52, no. 4: 30–37. 10.16382/j.cnki.1000-5579.2020.04.004.

[pchj70023-bib-0007] Gong, S. , and M. Ye . 2021. “The COVID‐19 Epidemic and College Students' National Identity.” Youth Studies 2021, no. 3: 53–62.

[pchj70023-bib-0008] He, J. , and Z. Li . 2020. “Measurement Invariance of the Sub‐Questionnaires of Chinese Physician‐Patient Social Mentality Questionnaire Across Gender.” Psychology: Techniques and Applications 8, no. 7: 406–413. 10.16842/j.cnki.issn2095-5588.2020.07.003.

[pchj70023-bib-0009] Heim, E. , H. Steinmetz , M. D. Zeigenfuse , A. Maercker , and J. Margraf . 2018. “The Circular Structure of Values: The Case of China.” International Journal of Psychology 53, no. 5: 339–348. 10.1002/ijop.12390. 27709607

[pchj70023-bib-0010] Hofstede, G. 1993. “Cultural Constraints in Management Theories.” Academy of Management Perspectives 7, no. 1: 81–94.

[pchj70023-bib-0011] Hou, J. 2017. “Evaluation of the Values of ‘benevolence, Righteousness, Propriety, Wisdom, and Trustworthiness’ and Their Modern Transformation.” People's Tribune 2017, no. 14: 138–139. 10.16619/j.cnki.rmlt.2017.14.060.

[pchj70023-bib-0012] Jiang, M. 2016. “Analysis of the Impact of Collectivist Culture on Cross‐Departmental Collaboration: A Comparative Perspective of Chinese and Western Cultures.” Social Sciences in Yunnan 2016, no. 4: 140–144.

[pchj70023-bib-0013] Jin, S. , L. Li , H. Che , and L. He . 2019. “The Characteristics of Chinese People's System of Values and It's Compatibility to Core Socialist Values.” Journal of Psychological Science 42, no. 3: 722–730. 10.16719/j.cnki.1671-6981.20190331.

[pchj70023-bib-0014] Lee, J. A. , S. Ye , J. N. Sneddon , P. R. Collins , and E. Daniel . 2017. “Does the Intra‐Individual Structure of Values Exist in Young Children?” Personality and Individual Differences 110: 125–130. 10.1016/j.paid.2017.01.038.

[pchj70023-bib-0015] Liu, P. , X. Wang , D. Li , R. Zhang , and J. Han . 2021. “The Benefits of Self‐Transcendence: Examining the Role of Values on Mental Health Among Adolescents Across Regions in China.” Frontiers in Psychology 12: 630420. 10.3389/fpsyg.2021.630420. 33679555 PMC7925830

[pchj70023-bib-0016] Lu, L. , and R. Gilmour . 2004. “Culture and Conceptions of Happiness: Individual Oriented and Social Oriented Swb.” Journal of Happiness Studies 5, no. 3: 269–291. 10.1007/s10902-004-8789-5.

[pchj70023-bib-0017] Maercker, A. , X. Chi Zhang , Z. Gao , et al. 2015. “Personal Value Orientations as Mediated Predictors of Mental Health: A Three‐Culture Study of Chinese, Russian, and German University Students.” International Journal of Clinical and Health Psychology 15, no. 1: 8–17. 10.1016/j.ijchp.2014.06.001. 30487817 PMC6224790

[pchj70023-bib-0018] Reise, S. P. , N. G. Waller , and A. L. Comrey . 2000. “Factor Analysis and Scale Revision.” Psychological Assessment 12: 287–297. 11021152 10.1037//1040-3590.12.3.287

[pchj70023-bib-0019] Sagiv, L. , S. Roccas , J. Cieciuch , and S. H. Schwartz . 2017. “Personal Values in Human Life.” Nature Human Behaviour 1, no. 9: 630–639. 10.1038/s41562-017-0185-3. 31024134

[pchj70023-bib-0020] Sagiv, L. , and S. H. Schwartz . 2021. “Personal Values Across Cultures.” Annual Review of Psychology 73: 517–546. 10.1146/annurev-psych-020821-125100. 34665670

[pchj70023-bib-0021] Schwartz, S. H. 1992. “Universals in the Content and Structure of Values: Theoretical Advances and Empirical Tests in 20 Countries.” Advances in Experimental Social Psychology, edited by M. P. Zanna , vol. 25, 1–65. Elsevier.

[pchj70023-bib-0022] Schwartz, S. H. 2006. “Les valeurs de base de la personne: théorie, mesures et applications.” Revue Française de Sociologie 47, no. 4: 929–968.

[pchj70023-bib-0023] Schwartz, S. H. 2012. “An Overview of the Schwartz Theory of Basic Values.” Online Readings in Psychology and Culture 2, no. 1: 11. 10.9707/2307-0919.1116.

[pchj70023-bib-0024] Schwartz, S. H. , and J. Cieciuch . 2021. “Measuring the Refined Theory of Individual Values in 49 Cultural Groups: Psychometrics of the Revised Portrait Value Questionnaire.” Assessment 29, no. 5: 1005–1019. 10.1177/1073191121998760. 33682477 PMC9131418

[pchj70023-bib-0025] Schwartz, S. H. , J. Cieciuch , M. Vecchione , et al. 2012. “Refining the Theory of Basic Individual Values.” Journal of Personality and Social Psychology 103, no. 4: 663–688. 10.1037/a0029393. 22823292

[pchj70023-bib-0026] Schwartz, S. H. , and F. Sortheix . 2018. Values and Subjective Well‐Being. DEF Publishers.

[pchj70023-bib-0027] Shao, F. 2017. “Research on Xi Jinping's Thought of Human Destiny Community and Contemporary Value.” Social Studies, no. 4: 1–8.

[pchj70023-bib-0028] Smetana, J. G. , N. Campione‐Barr , and A. Metzger . 2006. “Adolescent Development in Interpersonal and Societal Contexts.” Annual Review of Psychology 57: 255–284. 10.1146/annurev.psych.57.102904.190124. 16318596

[pchj70023-bib-0029] Steinmetz, H. , R. Isidor , and N. Baeuerle . 2012. “Testing the Circular Structure of Human Values: A Meta‐Analytical Structural Equation Modelling Approach.” Survey Research Methods 6: 61–75. 10.18148/srm/2012.v6i1.5096.

[pchj70023-bib-0030] ten Berge, J. M. 1986. “Rotation to Perfect Congruence and the Cross‐Validation of Component Weights Across Populations.” Multivariate Behavioral Research 21, no. 1: 41–64. 10.1207/s15327906mbr21013. 26760919

[pchj70023-bib-0031] The State Council of the People's Republic of China . 2013. Guidelines on the Cultivation and Practice of the Socialist Core Values. State Council of the People's Republic of China. https://www.gov.cn/zhengce/202203/content_3635148.htm.

[pchj70023-bib-0032] Wang, M. , and X. Bi . 2018. Latent Variable Modeling Using Mplus. Chongqing University Press.

[pchj70023-bib-0033] Wang, X. , N. Zhu , M. Wei , S. Chen , and Y. Liu . 2025. “The Relationship Between Content and Types of Personal Values and Depression in Chinese Adolescents.” Children and Youth Services Review 172: 108281. 10.1016/j.childyousth.2025.108281.

[pchj70023-bib-0034] Wang, Z. , and Y. You . 2013. “East Asian Adolescents' Democratic Attitudes and Democratic Behaviors: Generational Changes and Life Cycle Characteristics.” Open Times 2013, no. 6: 146–162. https://kns.cnki.net/kcms2/article/abstract?v=ZODoBDhYdpfTTg9XlwjO9fc20YBJU6L5Q6CKeOU5zM9z6nQK2a-jsqa3F9csUJfDB4W2h3ztxfsRvRJOkAkXuU9KcSK0vj1MaKaIB6SO7rRRfy6JySoHdjf_Knewiqev7kuMuA_Jgy-BphTb0T22Qxk5mldTrJXX4xBc1TN-ExmHr98DOIOwPRAWy9o5OivL&uniplatform=NZKPT&language=CHS.

[pchj70023-bib-0035] Xie, J.‐Q. , X.‐Q. Yin , J. Qiu , et al. 2023. “Latent Profile Analysis of Personal Values Among Chinese College Students: Associations With Mental Health Disorders and Life Satisfaction.” Current Psychology 42, no. 31: 27232–27244. 10.1007/s12144-022-03861-x. PMC9575634 36277265

[pchj70023-bib-0036] Zhai, X. 2004. “Favor, Face and Reproduction of the Power: A Way of Social Exchange in an Reasonableness Society.” Sociological Studies 2004, no. 5: 48–57. 10.19934/j.cnki.shxyj.2004.05.005.

[pchj70023-bib-0037] Zhang, K. 2015. “Basis of Social Governance: From Identity to Role.” Governance Studies 31, no. 5: 5–14. 10.15944/j.cnki.33-1010/d.2015.05.001.

[pchj70023-bib-0038] Zhong, S. , and J. Li . 2025. “The Generative Logic, Core Essence, and Practical Approach for Ideological‐Political Teachers to Keep Their Country in Mind.” Leading Journal of Ideological & Theoretical Education 2025, no. 1: 104–111. 10.16580/j.sxlljydk.2025.01.004.

